# SIRT1 is a Direct Coactivator of Thyroid Hormone Receptor β1 with Gene-Specific Actions

**DOI:** 10.1371/journal.pone.0070097

**Published:** 2013-07-26

**Authors:** Ji Ho Suh, Douglas H. Sieglaff, Aijun Zhang, Xuefeng Xia, Aleksandra Cvoro, Glenn E. Winnier, Paul Webb

**Affiliations:** Genomic Medicine Program, Methodist Hospital Research Institute, Houston, Texas, United States of America; University Claude Bernard Lyon 1, France

## Abstract

Sirtuin 1 (SIRT1) NAD^+^-dependent deacetylase regulates energy metabolism by modulating expression of genes involved in gluconeogenesis and other liver fasting responses. While many effects of SIRT1 on gene expression are mediated by deacetylation and activation of peroxisome proliferator activated receptor coactivator α (PGC-1α), SIRT1 also binds directly to DNA bound transcription factors, including nuclear receptors (NRs), to modulate their activity. Since thyroid hormone receptor β1 (TRβ1) regulates several SIRT1 target genes in liver and interacts with PGC-1α, we hypothesized that SIRT1 may influence TRβ1. Here, we confirm that SIRT1 cooperates with PGC-1α to enhance response to triiodothyronine, T_3_. We also find, however, that SIRT1 stimulates TRβ1 activity in a manner that is independent of PGC-1α but requires SIRT1 deacetylase activity. SIRT1 interacts with TRβ1 *in vitro*, promotes TRβ1 deacetylation in the presence of T_3_ and enhances ubiquitin-dependent TRβ1 turnover; a common response of NRs to activating ligands. More surprisingly, SIRT1 knockdown only strongly inhibits T_3_ response of a subset of TRβ1 target genes, including glucose 6 phosphatase (G-6-Pc), and this is associated with blockade of TRβ1 binding to the G-6-Pc promoter. Drugs that target the SIRT1 pathway, resveratrol and nicotinamide, modulate T_3_ response at dual TRβ1/SIRT1 target genes. We propose that SIRT1 is a gene-specific TRβ1 co-regulator and TRβ1/SIRT1 interactions could play important roles in regulation of liver metabolic response. Our results open possibilities for modulation of subsets of TR target genes with drugs that influence the SIRT1 pathway.

## Introduction

Thyroid hormone receptors (TRs) are members of the nuclear hormone receptor (NR) family [1]. Both TRs regulate gene transcription by binding to specific DNA sequences (thyroid hormone response elements, TREs) and nucleating formation of protein assemblies which, in turn, influence organization and post-translational modification of nearby chromatin and RNA polymerase II recruitment and processivity [2]–[4]. TRs, like other NRs, harbor a hormone-dependent docking surface that binds several general coregulators, including the steroid receptor coactivator (SRC) family, TR associated protein 220 (TRAP220) and others, which are required for target gene induction by active thyroid hormone, predominantly triiodothyronine (T_3_). Coregulators are recruited sequentially in a dynamic and ordered process and activated TRs are eventually ubiquitinated and channeled into proteasomal degradation pathways [5], [6].

TR transcription complexes must also integrate responses to T_3_ signals with those of other signaling pathways. TRs cooperate with particular subsets of heterologous DNA bound transcription factors (TFs) in composite modules, including CTCF [7] and SREBP1 [8], and particular TR/TF combinations could be responsible for integration of different signaling pathways. TR coregulators could also play a role. For example, peroxisome proliferator activated receptor γ coactivator 1α (PGC-1α) is induced in different tissues in response to a variety of different environmental signals and strongly potentiates TR activity [9]–[15]. In liver, PGC-1α is induced under fasting conditions and is required for optimal T_3_ activation of genes involved in fatty acid β-oxidation, mitochondrial activity and other metabolic pathways [13]–[15].

Sirtuin 1 (SIRT1) is an NAD^+^-dependent deacetylase that is activated by resveratrol and regulates the expression of genes involved in fasting response and resistance to metabolic diseases [16], [17]. SIRT1 regulates activity of several TFs that bind to PGC-1α via targeted deacetylation of multiple PGC-1α lysine residues and enhancement of PGC-1α activity [18]. For example, SIRT1 enhances the activity of the NR PPARα in liver through PGC-1α [19]. In addition, SIRT1 binds directly to DNA bound TFs, including NRs, to influence TF activity [20]–[22]. For example, SIRT1 enhances the response of liver X receptor (LXR) α to agonists and this is accompanied by LXR deacetylation and SIRT1-dependent channeling of active LXRα into ubiquitin-dependent degradation pathways [20].

There are superficial overlaps between the actions of TRs and SIRT1 in liver [16], [23]; both factors exert similar effects upon genes involved in gluconeogenesis, fatty acid oxidation and mitochondrial function. Since TR binds PGC-1α [9], we tested the possibility that SIRT1 may enhance activity of TRβ1, the predominant TR subtype in liver, via effects upon PGC-1α activity. We confirm that SIRT1 synergizes with PGC-1α to potentiate T_3_ response, in accordance with recent findings of another group [24], but also find that SIRT1 enhances TRβ1 activity independently of PGC-1α. More surprisingly, requirements for SIRT1 in T_3_ response are highly gene-specific and, in one case, associated with hormone and SIRT1-dependent transcription complex assembly on DNA. We propose that TRβ1/SIRT1 complex formation may serve as a checkpoint for regulation of key genes with important roles in metabolic response and our results open possibilities for modulation of subsets of T_3_ dependent genes with drugs that target the SIRT1 pathway.

## Materials and Methods

### Plasmids and Reagents

Mammalian expression vectors for human TRβ1 and PGC-1α and the bacterial expression vector for TRβ1 (GST-TRβ1) were described previously [9]. SIRT1 were cloned by PCR from a cDNA library of HepG2 cells in frame at BamHI and XbaI sites of pCDNA3Flag vector. The mammalian expression vectors for mouse SIRT1 wild type and SIRT1 mutant (H355Y) were provided as gifts from Dr. Tadahiro Kitamura (Gunma University). The DR-4-luc reporter plasmid was described previously [9]. The G-6-Pc-Luc reporter plasmid was constructed by cloning a G-6-Pc promoter region (−2785 to −2358) into vector pGL4 basic (Promega). G-6-Pc promoter was generated by PCR amplification using two primer sets: forward, 5′-CGGTACCTGAGCTCGCTAGCCCTTTGAGAATCCACGGTGT-3′, and reverse, 5′- AAGCTTGGCCGCCGAGACCCTAACACTTGGGTCACG-3′. The PCK1-Luc reporter plasmid was constructed by cloning a TRE located 13kb downstream from the 3′ end of PCK1 (+18,719 to +19,603, +1 being the A base in the translation initiation codon) into vector pGL4.23 (Promega). The PCK1 promoter was generated by PCR amplification using two primer sets: forward, 5′-CGGTACCTGAGCTCGCTAGCAACAATAGTGGCAGGCATCC-3′, and reverse, 5′-AAGCTTGGCCGCCGAGCCCTCCACAGAAGTCCACAT-3′. The method for reporter construction was described previously [25]. SLC16A6-Luc (S710535) and MYH6-Luc (S704901) reporter plasmids were purchased from Switchgear Genomics.

Resveratrol (R5010), Nicotinamide (N3376), and MG-132 (C2211) were purchased from Sigma Aldrich.

### Cell Culture and Transient Transfection

HepG2 cells stably expressing Flag tagged TRβ1 were described previously [26]. 293T cells were purchased from ATCC (CRL-11268). 293T cells and Flag tagged TRβ1 overexpressed HepG2 cells were maintained in Dulbecco’s modified Eagle’s medium (DMEM) supplemented with 10% fetal bovine serum. Cells were plated in 24-well plates and transfected with expression plasmids, reporter plasmid, and control *lacZ* expression plasmid pCMV-β, by using Fugene HD Transfection reagent (Roche) according to manufacturer’s instructions. Total amounts of expression vector plasmids were kept constant by the addition of appropriate amounts of empty pCMV vector. Cells maintained in 10% T_3_-stripped serum were treated with 10 nM triiodothyronine (T_3_) for 24 h following transfection. Resveratrol or nicotinamide was added for 6 or 24 h prior to harvest. Luciferase and β-galactosidase activities were assayed as described [9], [25].

### Coimmunoprecipitation and Western Blot

Coimmunoprecipitations were performed from extracts of 293T cells that were cotransfected with 1 µg of Myc tagged TRβ1 and Flag tagged SIRT1 expression plasmids. Cotransfected 293T Cells or Flag tagged TRβ1 expressing HepG2 were treated +/− 10 nM T_3_ for 12 h following transfection, and harvested in RIPA cell lysis buffer (50 mM Tris-HCl, pH 7.5, 150 mM NaCl, 2.5 mM EGTA, 1% NP-40, Protease inhibitor cocktail (Roche). Whole-cell lysate (400 µg) was incubated with 2 µg of anti-Flag (A2220) or anti-myc (A7470) antibody conjugated agarose bead slurry (Sigma Aldrich) for 12 h at 4°C. Antibody conjugated agarose beads were washed three times with RIPA buffer at 4°C, and bound proteins were separated by SDS-PAGE. Proteins were transferred to a PVDF membrane (Bio-Rad), subjected to Western blot analysis with anti-Myc (Sigma Aldrich M4439), anti-Flag (Cell Signaling #2044), anti-SIRT1 (Cell Signaling #2496), anti-TRβ1 (Santa Cruz Biotechnology sc-738) and anti-GAPDH (Cell Signaling #2118), and then detected with an ECL kit (Amersham Pharmacia). Anti-acetylated-Lysine (Cell Signaling #9441) and anti-ubiquitin (Millipore MAB1510) antibodies were used to detect acetylation and ubiquitination of TRβ1. Anti-PGC-1α antibody were purchased from Santa Cruz Biotechnology (sc-13067).

### GST Pull-down

GST and GST-TRβ1 fusions were expressed in *Escherichia coli* BL21 cells and isolated with glutathione-Sepharose-4B beads (GE Healthcare Life Sciences). Immobilized GST fusions were then incubated with SIRT1 protein produced by *in vitro* translation using the TNT-coupled transcription-translation system (Promega). Binding reactions were carried out in 250 µl of GST binding buffer (20 mM Tris-HCl, pH 7.9, 100 mM NaCl, 10 % glycerol, 0.05 % NP-40, 5 mM MgCl_2_, 0.5 mM EDTA, 1 mM dithiothreitol, and 1.5 % bovine serum albumin) for 4 h at 4°C. The beads were washed three times with 1 ml of GST binding buffer. Bound proteins were eluted by the addition of 20 µl of SDS loading buffer, and were analyzed by Western blot analysis using anti-SIRT1 antibody. *Escherichia coli* BL21 cells were purchased from Invitrogen.

### Adenovirus Infection

Adenoviruses that express mouse wild type or mutant (H355Y) SIRT1 were kindly provided as gifts from Dr. Tadahiro Kitamura (Gunma University). Flag tagged TRβ1 overexpressing HepG2 cells were infected with adenovirus expressing SIRT1 (AdSIRT1) or null adenovirus at multiplicity of infection = 50 and treated +/− T_3_.

### siRNA

SiRNAs for SIRT1 (ID no. HSS177403, HSS177404 and HSS118729) and PGC-1α (ID no. HSS116797, HSS116798, HSS116799) were purchased from Invitrogen Life Technologies. HepG2-TRβ1 cells were transfected with siRNA (100 nM) using Lipofectamine RNAiMAX reagent (Invitrogen Life Technologies) according to manufacturer’s instructions.

### qPCR

Real-time PCR was performed as described previously [26], using the Roche LightCycler 480 RT PCR machine and SYBR Green Mastermix (Roche) according to the manufacturer’s procedure. Sequences of primers used for Real-time PCR are available upon request. Relative mRNA levels were calculated by comparative the cycle threshold method using GAPDH as the internal control. GAPDH level was not affected by T_3_.

### Microarray Hybridization and Analysis

HumanHT-12 v4 whole genome expression arrays were purchased from Illumina. cRNA synthesis and labeling were performed using Illumina® TotalPrep™-96 RNA Amplification Kit (Ambion). Labeling in vitro transcription reaction was performed at 37°C for 14 h. Biotinylated cRNA samples were hybridized to arrays at 58°C for 18 h according to manufacturer’s protocol. Arrays were scanned using BeadArray Reader. Unmodified microarray data obtained from GenomeStudio was background-subtracted and quantile-normalized using the lumi package [35] and analyzed with the limma package [36] within R [37]. All analysis was corrected for multiple hypotheses testing [38], and effects determined to be significant when 2-fold with an adjusted p-value 0.05. To facilitate comparisons among the various datasets, all data was uploaded into a SQLite3 database [39]. Heatmaps were produced and clustered using multiarray viewer [40].

To determine T_3_-responsive gene transcripts affected by SIRT1 KD, we compared the T_3_-response in the NC-siRNA against SIRT1 siRNA through pattern analysis as done previously (26). In brief, including the three affects (T_3_-response in NC-siRNA, T3-response in SIRT1 siRNA, and SIRT siRNA in the absence of T_3_) a 3-by-3 permutation of down-regulated (0.5) and up-regulated (2) and no change (1) results in 27 possible patterns (supplemental). The Euclidean distance between experimental data and the 27 patterns were calculated, with the matching pattern receiving the minimal Euclidean distance.

### Chromatin Immunoprecipitation (ChIP) Assay

Flag tagged TRβ1 overexpressed HepG2 cells were transfected with SIRT1 siRNA or control siRNA and treated +/− 10 nM T_3_ for 24 h following transfection. ChIP assays were performed by SimpleChip Enzymatic Chromatin IP Kit (Cell Signaling) according to manufacturer’s instructions. 10 % (v/v) of the supernatant was saved as ‘input’ chromatin prior to immunoprecipitation. Anti-Flag antibody conjugated agarose bead slurry (Sigma Aldrich A2220) and anti-SIRT1 (Cell Signaling #2496) were used for immunoprecipitation. Immunoprecipitated DNA and input-sheared DNA were subjected to PCR using a primer pair for G-6-Pc (forward, 5′-GAGGCGTCTCAGAAAACAGG-3′, and reverse, 5′-GCAGTGACCTCTGGGATGAG-3′) or for PCK1 (forward, 5′-AGTTTCTCCTCCTCCTGCAGACAA-3′; and reverse, 5′- AGGACCTGACCAGAAGTCAGAACA-3′), which amplify regions spanning the thyroid response element (TRE). IgG was used as an immunoprecipitation control.

### Microarray

Flag tagged TRβ1 overexpressed HepG2 cells were transfected with control siRNA (100 nM) or SIRT1 siRNA (100 nM) and treated +/− T_3_. Hybridizations and microarrays were performed as described previously.

### Statistical Analysis

All results are the means ± SD. Statistical analysis was performed using GraphPad Prism software (GraphPad Inc., San Diego, CA). Comparisons of groups were performed using a Student’s t test.

P < 0.05 was considered statistically significant. All experiments were performed at least three times.

## Results

### SIRT1 Enhances TRβ1 Activity in PGC-1α Dependent and Independent Manners

**Figure 1:**
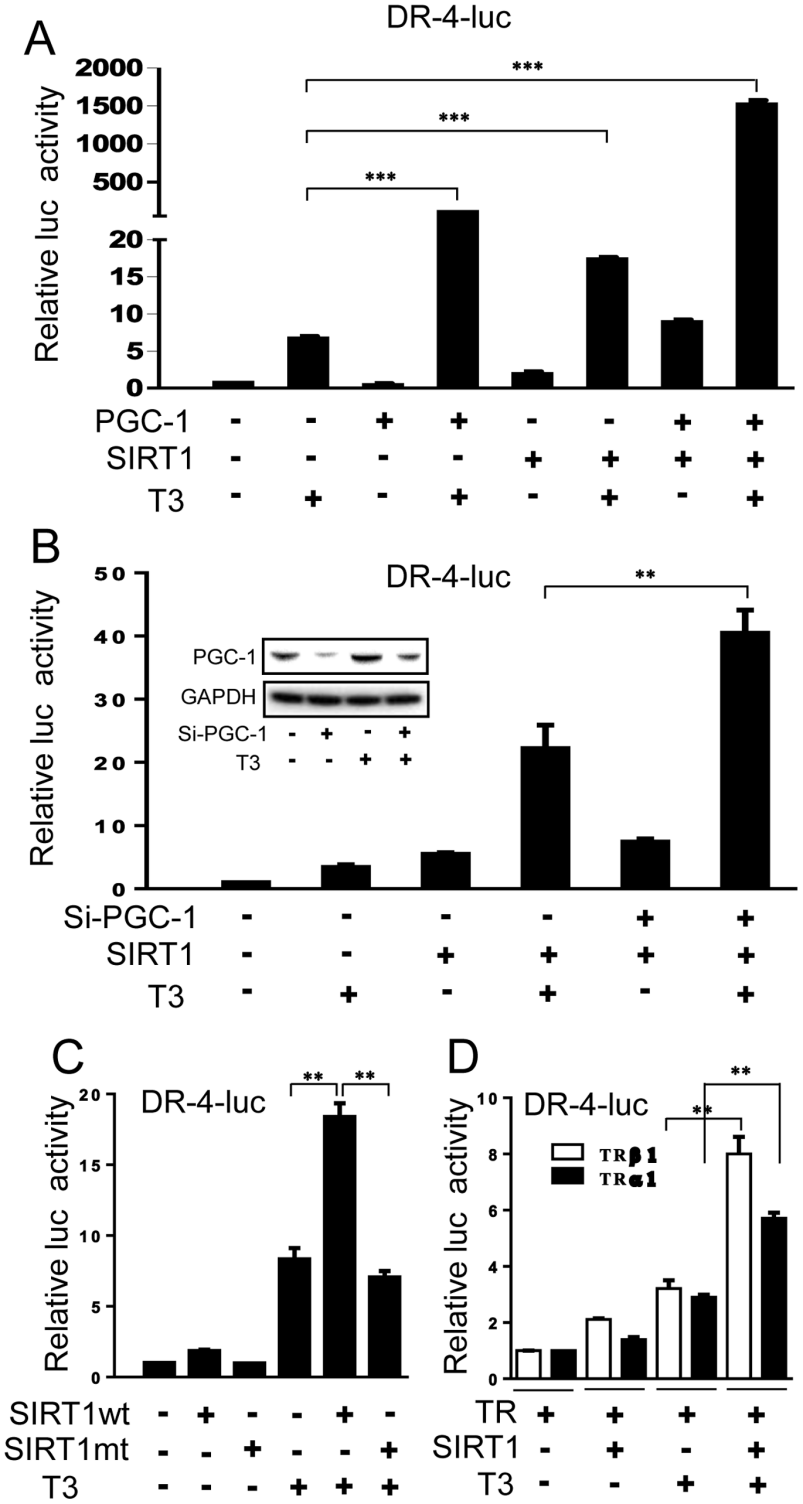
SIRT1 is a TRβ1 co-regulator. (**A**) Graph representing luciferase activity measured in extracts of HepG2 TRβ1 cells transfected with DR-4-luc and expression vectors for PGC-1α, SIRT1 or both and treated +/− T_3_. (**B**) Results of luciferase assay in HepG2 TRβ cells when siRNA against PGC-1α is used. Inset represents a western blot of cell extracts using PGC-1α antibody to confirm PGC-1α knockdown. (**C**) As in Fig. 1B, but using expression vectors for wild type SIRT1 and deacetylase defective mutant of SIRT1 (H355Y). (**D**) Luciferase activity measured in 293T cells transfected with expression vectors for TRβ1 or TRα +/− SIRT1 expression vector and treated +/− T_3_. The levels of luciferase activity were normalized to lacZ expression. All data are representative of at least three independent experiments with similar results. All values represent the mean ± SD of duplicate samples. ***, *P* < 0.001; **, *P* < 0.01.

To evaluate the possibility that SIRT1 modulates TRβ1 activity, we examined SIRT1 effects on T_3_ response at a standard T_3_ inducible reporter driven by a TRE composed of a direct repeat of the consensus TR binding half-site AGGTCA (DR-4) [27]. We transfected the DR-4 reporter into HepG2 liver cells that stably express TRβ1 +/− expression vectors for PGC-1α and SIRT1 and measured T_3_ effects on luciferase activity (Fig. 1A). As expected, T_3_ response was enhanced approximately 20-fold by PGC-1α whereas SIRT1 increased T_3_ response 2.5 fold. Cotransfection of SIRT1 and PGC-1α resulted in synergistic increases in TRβ1 activity; together, SIRT1 and PGC-1α potentiated T_3_ response by greater than 200-fold, consistent with the notion that SIRT1 cooperates with PGC-1α to stimulate TRβ1 activity [24]. Interestingly, SIRT1 overexpression had no obvious influence on acetylation of PGC-1α in these conditions, suggesting that SIRT1 must exert additional effects upon activity of the TRβ1/PGC-1α complex (Fig. S1).

We next examined effects of knockdown of endogenous PGC-1α expression in the HepG2-TRβ cells (Fig. 1B). PGC-1α protein levels were greatly diminished after transfection of PGC-1α siRNA, as determined by western analysis of cell extracts (Fig. 1B, inset). Under these conditions, however, SIRT1 coactivation of TRβ1 was unaffected, and even, partially enhanced. Thus, SIRT1-dependent enhancement of T_3_ response is independent of PGC-1α in this assay. SIRT1 enzymatic activity was required for TRβ1 coactivation; a deacetylase defective mutant (H355Y) of SIRT1 did not enhance T_3_ response (Fig. 1C). We also verified that SIRT1 enhanced activity of both TR subtypes in transfections into 293T cells, which do not express endogenous TRs, albeit with a modest preference for TRβ1 versus TRα1 (Fig. 1D).

### SIRT1 Interacts with TRβ1

**Figure 2:**
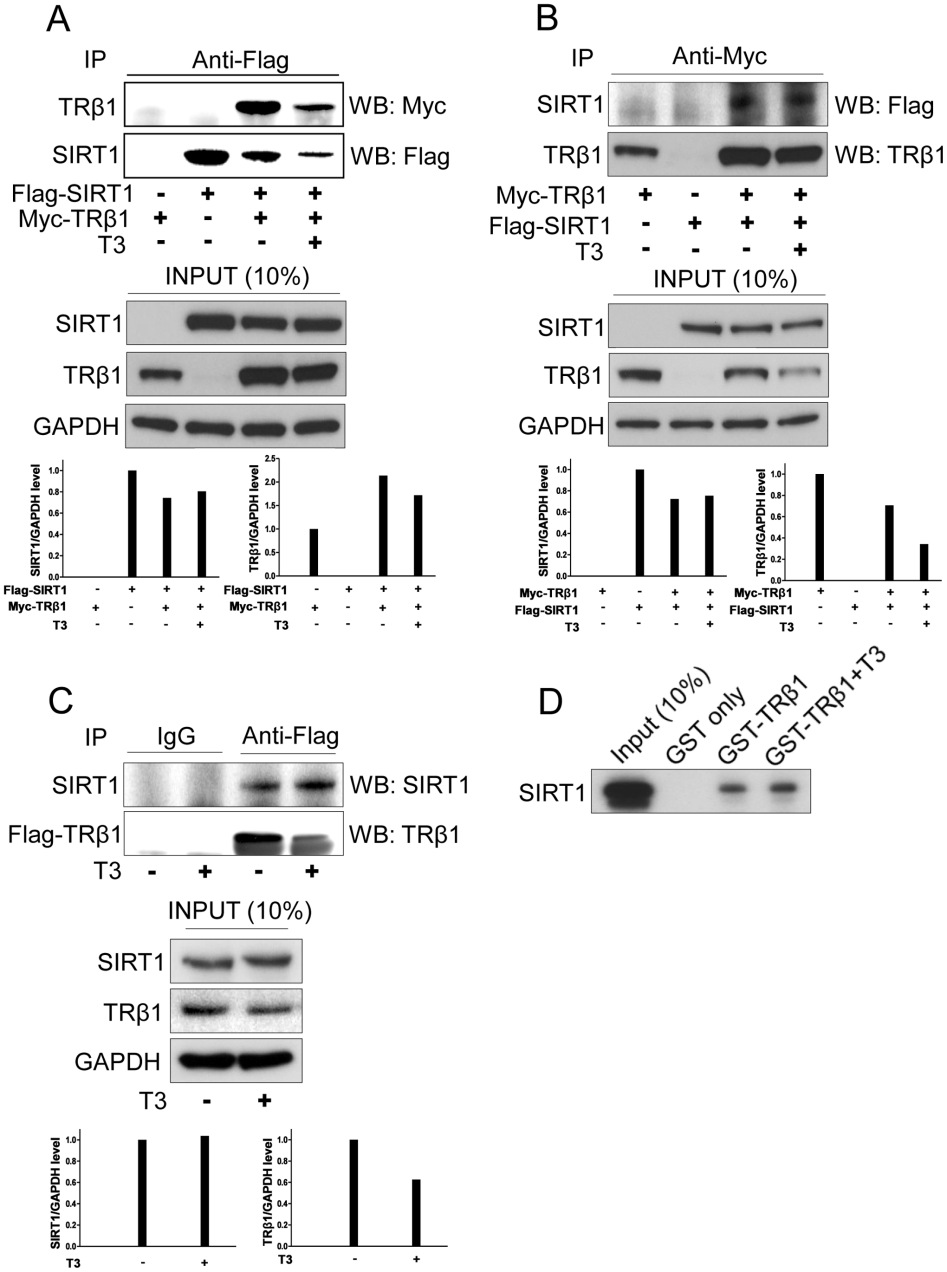
SIRT1 interacts with TRβ1. **(A, B)** Co-immunoprecipitation assays from 293T cells transfected with expression vectors for Flag tagged SIRT1 and Myc tagged TRβ1 and treated +/− 10 nM T_3_ for 12 hr. Antibody used for immunoprecipitation is indicated at the top of the panel and antibody used for western analysis is indicated at the right hand side. Panels below represent western blots of input proteins or GAPDH loading control and quantitative scans of amounts of each protein detected in western analysis of input protein panels. (**C**) Co-immunoprecipitation assays from HepG2 cells which stably express Flag tagged TRβ1. TRβ1 was immunoprecipitated with anti-flag and western analysis of precipitants was performed with antibodies indicated at the right of each panel. Lower panels represent western blots of input proteins or loading control. (**D**) GST pull-down assays to demonstrate SIRT1 directly interacts with TRβ1 *in vitro*. The image represents a western blot of an SDS-PAGE gel used to separate input and retained SIRT1 after binding reaction with GST- or GST- full length TRβ1 fusions linked to a solid support and probed with SIRT1 antibody. Input represents 10% of the total volume of SIRT1 used in the binding assay.

To investigate whether SIRT1 interacts with TRβ1, we performed co-immunoprecipitations from extracts of 293 cells transfected with affinity tagged versions of SIRT1 (flag) and TRβ1 (myc) to qualitatively assess SIRT1/TRβ1 interactions in different conditions. SIRT1 precipitation with anti-flag antibody resulted in co-precipitation of myc-tagged TRβ1 (Fig. 2A). Conversely, TRβ1 precipitation with anti-myc antibody resulted in co-precipitation of SIRT1 (Fig. 2B). Within this context, SIRT1/TRβ1 interactions appeared unaffected by T_3_. We also performed co-immunoprecipitation assays in the HepG2 stably expressing TRβ1 and showed that endogenous SIRT1 co-precipitated with TRβ1 and, again, this effect was ligand-independent (Fig. 2C).

To test whether SIRT1 interacts with TRβ1 *in vitro*, we performed glutathione-S-transferase (GST) pull-downs with purified bacterially expressed TRβ1. As shown in Fig. 2D, ^35^S-Methionine-labeled SIRT1 protein was retained by GST-TRβ1, but not by GST protein, in the absence and presence of T_3_. Thus, SIRT1 directly interacts with TRβ1 in a ligand-independent manner.

### SIRT1 Deacetylates TRβ1 and Down-regulates TRβ1 Protein Levels

**Figure 3:**
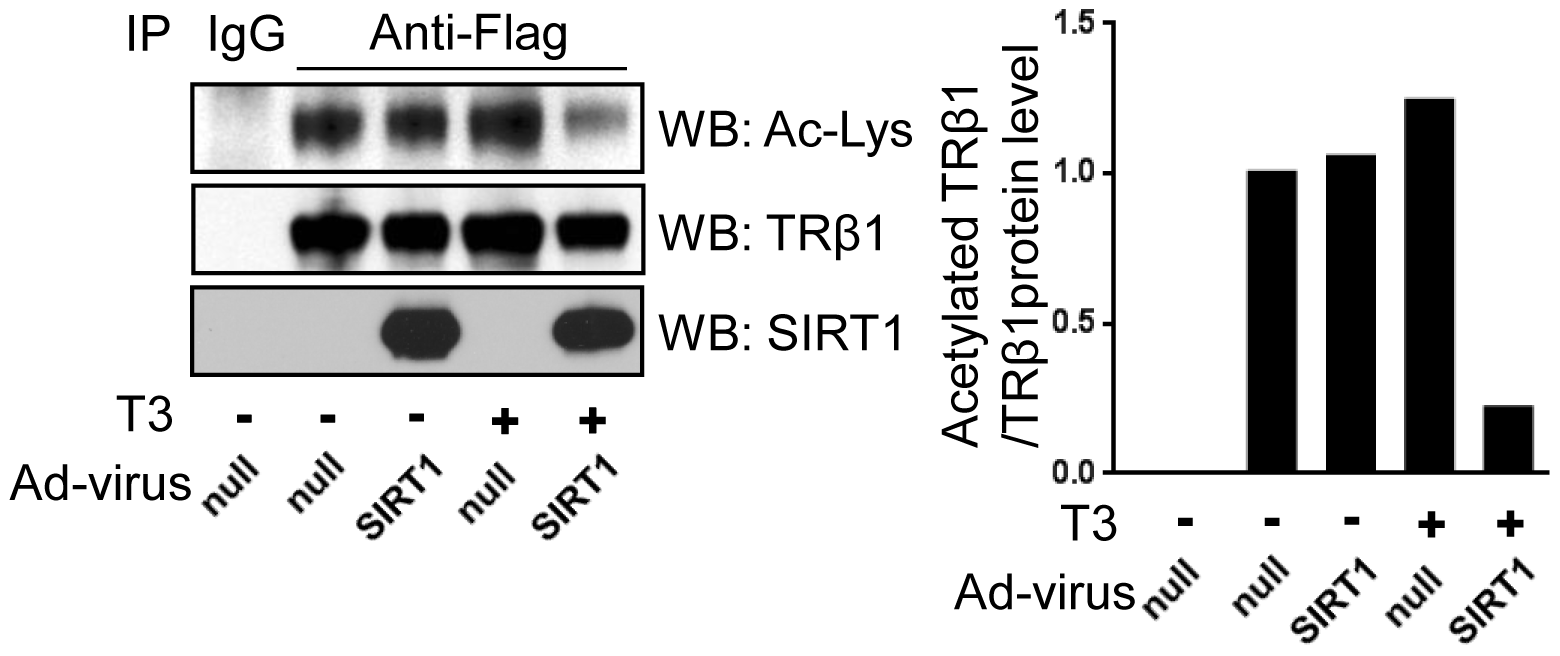
SIRT1 deacetylates TRβ1. Immunoprecipitation analysis of HepG2-TRβ1 cells infected with null adenovirus control or adSIRT1 and treated +/− T_3_. TRβ1 was immunoprecipitated with anti-Flag antibodies and precipitates were blotted with anti-acetyl-lysine, TRβ1 or SIRT1 antibodies. IgG control precipitation is shown at right. Acetylated TRβ1 levels relative to total TRβ1 were quantified by Phosphor Imager (right panel).

To determine whether activity SIRT1 influences TRβ1 acetylation state, we infected HepG2-TRβ1 cells with an adenovirus expressing SIRT1 (adSIRT1) and treated with T_3_ and immunoprecipitated TRβ1. Western blot analysis with an acetyl lysine specific antibody (Fig. 3) confirmed that TRβ1 is acetylated [28], [29] and also reveals that acetylation levels were specifically decreased in cells which overexpress SIRT1 and were treated with T_3_ (Fig. 3).

**Figure 4:**
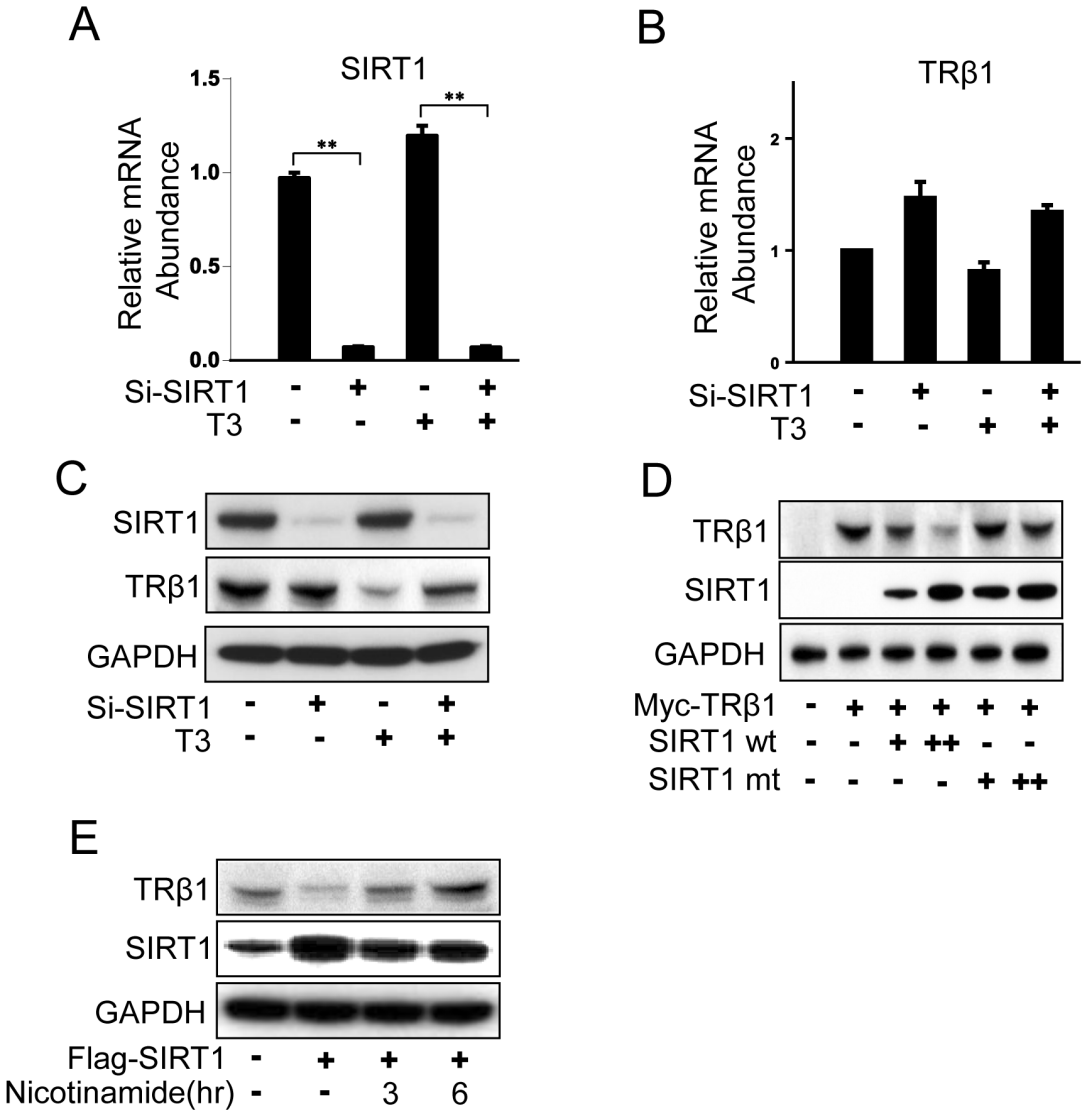
SIRT1 knockdown reverses hormone-dependent reduction of TRβ1 steady state levels. **(A, B)** qPCR analysis of endogenous SIRT1 mRNA and TRβ1 mRNA in HepG2-TRβ1 cells +/− transfection of SIRT1 siRNA. All data are representative of at least three independent experiments with similar results. All values represent the mean ± SD of duplicate samples. **, *P* < 0.01. (**C**) Western analysis of SIRT1 and TRβ1 protein levels in HepG2-TRβ1 cells +/− SIRT1 siRNA. Note that SIRT1 levels were strongly inhibited by SIRT1 siRNA treatment and that TRβ1 levels were unaffected in the absence of ligand, but that SIRT1 reversed T_3_-dependent reductions in TRβ1 protein levels. The lower panel represents GAPDH loading control for Western blot analyses. (**D**) SIRT1-dependent reduction of TRβ1 protein levels requires SIRT1 deacetylase activity. Western analysis of 293T cells transfected with TRβ1 +/− wild type or mutant (H355Y) SIRT1 expression vectors and treated with T_3_. (**E**) T_3_-dependent reductions in TRβ1 protein levels were reversed by nicotinamide. Panels show western analysis of HepG2-TRβ1 cell extracts transfected +/− SIRT1 expression vector and treated with T_3_ for 24hrs and 10 mM nicotinamide for indicated times. Note the recovery in TRβ1 levels after nicotinamide treatment. The lowest panel represents a western blot with anti-GAPDH antibody as loading control.

To determine how SIRT1 affects TRβ1 protein levels, we decreased SIRT1 levels using a specific SIRT1 siRNA in HepG2-TRβ cells. This treatment reduced SIRT1 mRNA and protein levels by more than 90% with no effect upon TRβ1 mRNA levels (Fig. 4A-C). TRβ1 protein levels were unaffected by SIRT1 knockdown in the absence of T_3_, however, there were T_3_-dependent reductions of TRβ1 steady state levels and these were partially reversed in the presence of the SIRT1 siRNA (Fig. 4C). Hormone activation usually results in diminished steady state levels of nuclear receptors and this phenomenon reflects increased ubiquitin-dependent receptor turnover; an essential step for renewal of NR transcription complexes [5]. Over the course of the study, T_3_ consistently reduced steady state TRβ levels, although the extent of this effect varied from modest (20%) to large (>80%). In this case, effects were large and we also observed that this effect was partly dependent upon SIRT1 enzymatic activity; wild type SIRT1 potentiated T_3_-dependent decreases in TRβ1 levels in transfected 293T cells but no decrease was observed in the presence of the deacetylase-defective mutant of SIRT1 (Fig. 4D). Nicotinamide, a SIRT1 inhibitor, also reversed T_3_-dependent reductions in TRβ1 levels in this cell type (Fig. 4E). This is consistent with the idea that SIRT1 activity is required for hormone-dependent reductions in TRβ1 steady state levels in these conditions, although nicotinamide may also slightly reduce SIRT1 levels and this could also contribute to this effect. We conclude that SIRT1 enhances T_3_-dependent reductions in TRβ1 steady state levels.

**Figure 5:**
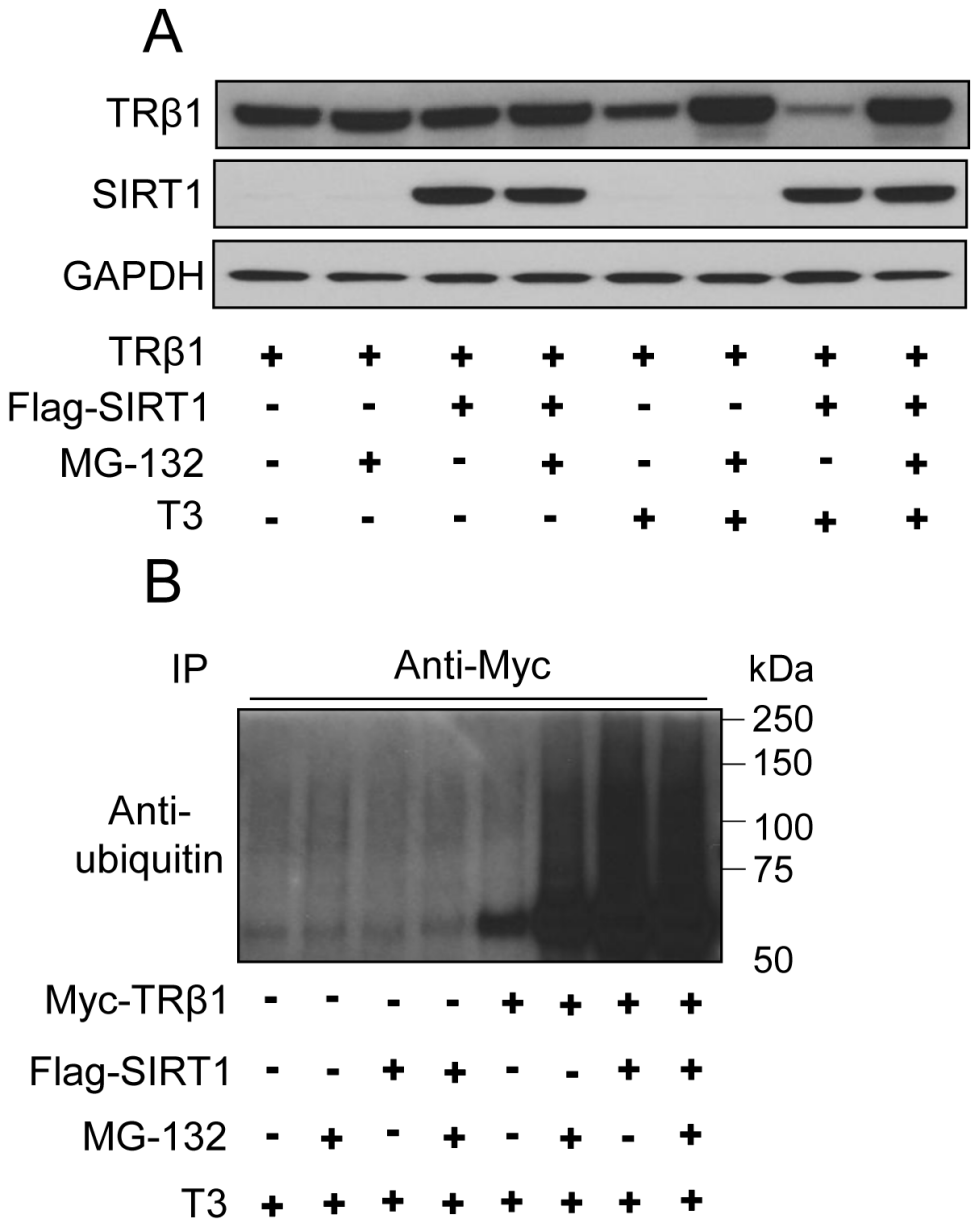
SIRT1 induces proteasome-dependent TRβ1 degradation and ubiquitination. (**A**) Western analysis of 293T cells transfected with myc-TRβ1 and SIRT1 expression vectors and treated with T_3_ for 24 hours and 20 µM MG-132 for 6 hours. Note the MG132-dependent increase in TRβ1 levels observed with SIRT1 and T_3_. The lower panel represents GAPDH loading control. (**B**) SIRT1 leads to ubiquitination of TRβ1 protein. The panel represents western analysis of extracts of 293T cells transfected with myc-TRβ1 and SIRT1 expression vectors and treated with T_3_ for 24 hours and 20 µM MG-132 for 6 hour, immunoprecipitated with anti-myc antibody and blotted with anti-ubiquitin antibody.

Since previous studies suggested that SIRT1 dependent de-acetylation of LXRs is associated with enhanced ubiquitin-dependent receptor turnover [20], we examined effects of inhibition of proteasome activity upon TRβ1 steady state levels and ubiquitination. In the absence of T_3_, TRβ1 protein levels were not changed by SIRT1 overexpression, treatment of proteasome inhibitor (MG132), or both in transfected 293T cells (Fig. 5A). In the presence of T_3_, however, treatment with MG132 led to increases in TRβ1 levels relative to that seen with overexpressed SIRT1 and T_3_ (Fig. 5A). Overexpression of SIRT1 was also associated with accumulation of higher molecular weight ubiquitinated forms of TRβ1, similar to that seen in the presence of proteasome inhibitor MG132 (Fig. 5B). Thus, SIRT1 overexpression results in T_3_-dependent deacetylation of TRβ1 and enhanced proteasome-mediated degradation and ubiquitination of TRβ1.

### SIRT1 Influences Expression of a Subset of TRβ1 Target Genes

**Figure 6:**
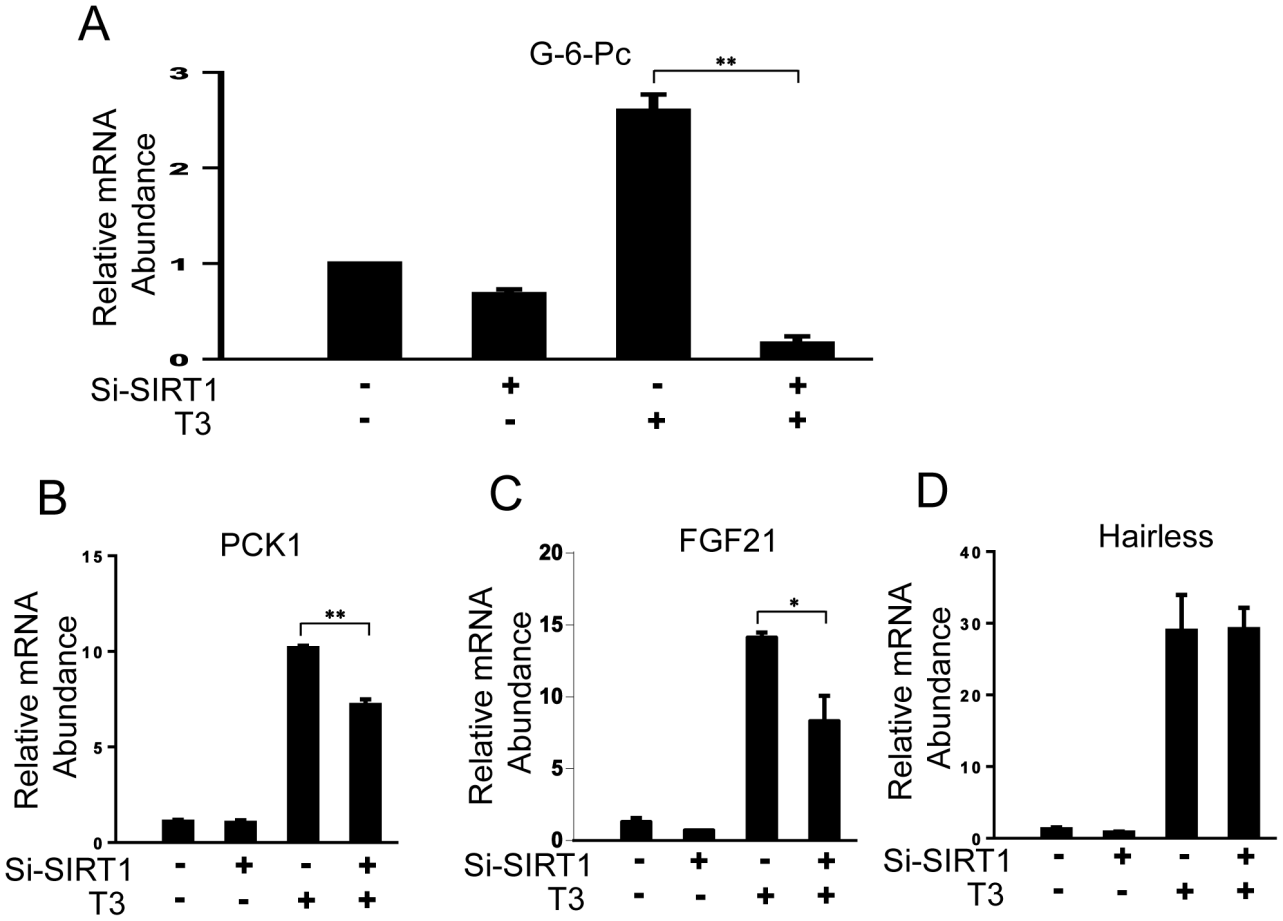
SIRT1 knockdown inhibits some TRβ1 target genes. (A–D) qPCR analysis of HepG2-TRβ1 cells extracts treated +/− T_3_ and SIRT1 siRNA. G-6-Pc (A), PCK1 (B), FGF21 (C) and Hairless HR (D). The data are representative of at least three independent experiments. All values represent the mean ± SD of duplicate samples. **, *P* < 0.01; *, *P* < 0.05.

To understand how knockdown of SIRT1 would influence expression of TRβ1 target genes involved in fasting responses, we examined effects of transfected SIRT1 siRNA on T_3_ response in HepG2-TRβ cells using qPCR analysis of selected TRβ1 targets [26]. SIRT1 siRNA treatment led to complete loss of T_3_ induction of the glucose 6 phosphatase (G-6-Pc) gene, which encodes an enzyme that catalyzes a key rate limiting step in gluconeogenesis (Fig. 6A). In fact, T_3_ suppressed G-6-Pc mRNA levels in the presence of SIRT1 siRNA relative to basal levels seen with SIRT1 siRNA alone. SIRT1 also modestly inhibited T_3_ induction of two other fasting response genes, phosphoenol pyruvate carboxykinase 1 (PCK1) and fibroblast growth factor 1 (FGF21) (Fig. 6B, C). However, T_3_ induction of other target genes, including Hairless (HR; Fig. 6D), was completely unaffected by SIRT1 knockdown. PGC-1α knockdown only slightly decreased mRNA level of these target genes, G-6-Pc and PCK1 (Fig. S3). This finding strengthens the suggestion that SIRT1-dependent regulation of these genes is independent of PGC-1α in these assay conditions.

**Figure 7:**
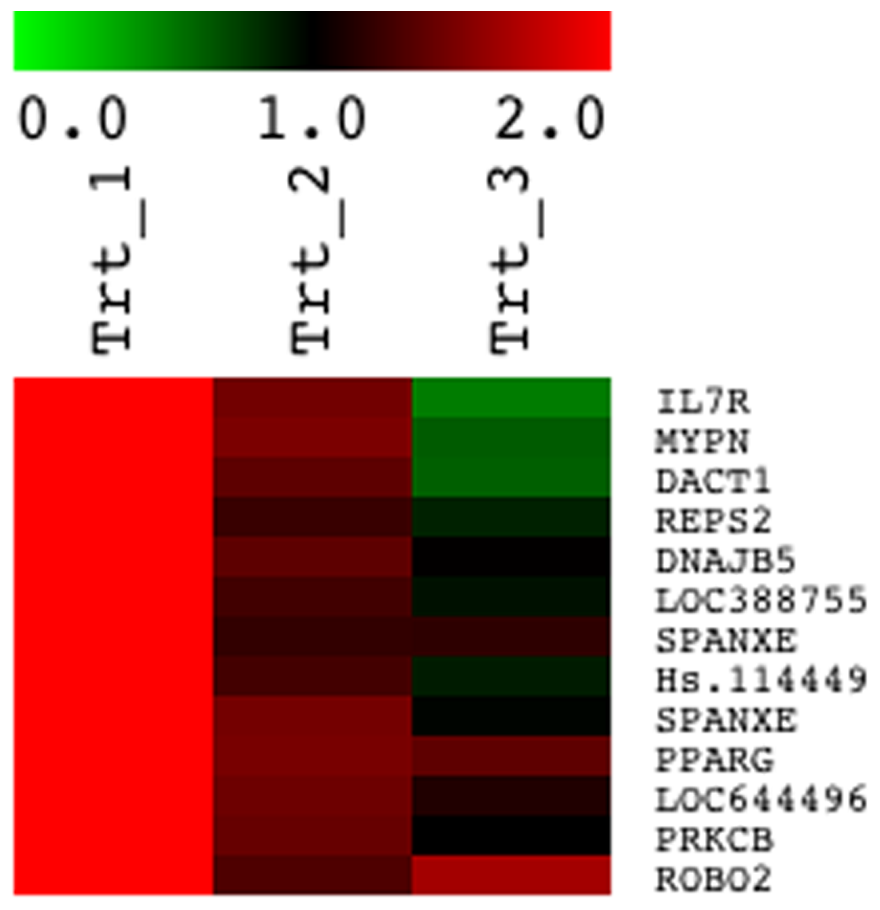
A subset of TRβ1 target genes that are inhibited by SIRT1 knockdown. Heat map representing results of array analysis performed on HepG2-TRβ1 cells treated +/− T_3_ and SIRT1 siRNA and displaying probe sets in which T_3_ response is inhibited by SIRT1 siRNA. Scale is shown at top. The first lane (1) represents T_3_ responses obtained in the presence of control siRNA, second lane (2) represents T_3_ responses obtained in the presence of SIRT1 siRNA. The third lane (3) represents comparison of mRNA expression levels in the presence of control siRNA or SIRT1 siRNA. Note that, in most instances in which SIRT1 inhibits these T_3_ responses, this effect is not accompanied by SIRT1-dependent changes in basal gene expression.

To explore the influences of SIRT1 knockdown upon T_3_ response more fully, we examined T_3_ induction +/− SIRT1 siRNA in HepG2-TRβ1 cells using an array based assay. As we documented previously [26], hundreds of genes displayed significant T_3_ response in this cell type, with most induced by T_3_ and a minority repressed (Fig. S2). However, SIRT1 siRNA treatment only inhibited T_3_ induction of a small subset of these TRβ1 target genes (Fig. 7) with the vast majority unaffected by SIRT1 (Fig. S2). There is no obvious defined functional association among the affected genes (i.e., functional ontology; not shown). Thus, SIRT1 is absolutely required for T_3_-induction of a very limited number of genes.

### SIRT1 Potentiates TRβ1 Activity at Native Regulatory Elements

**Figure 8:**
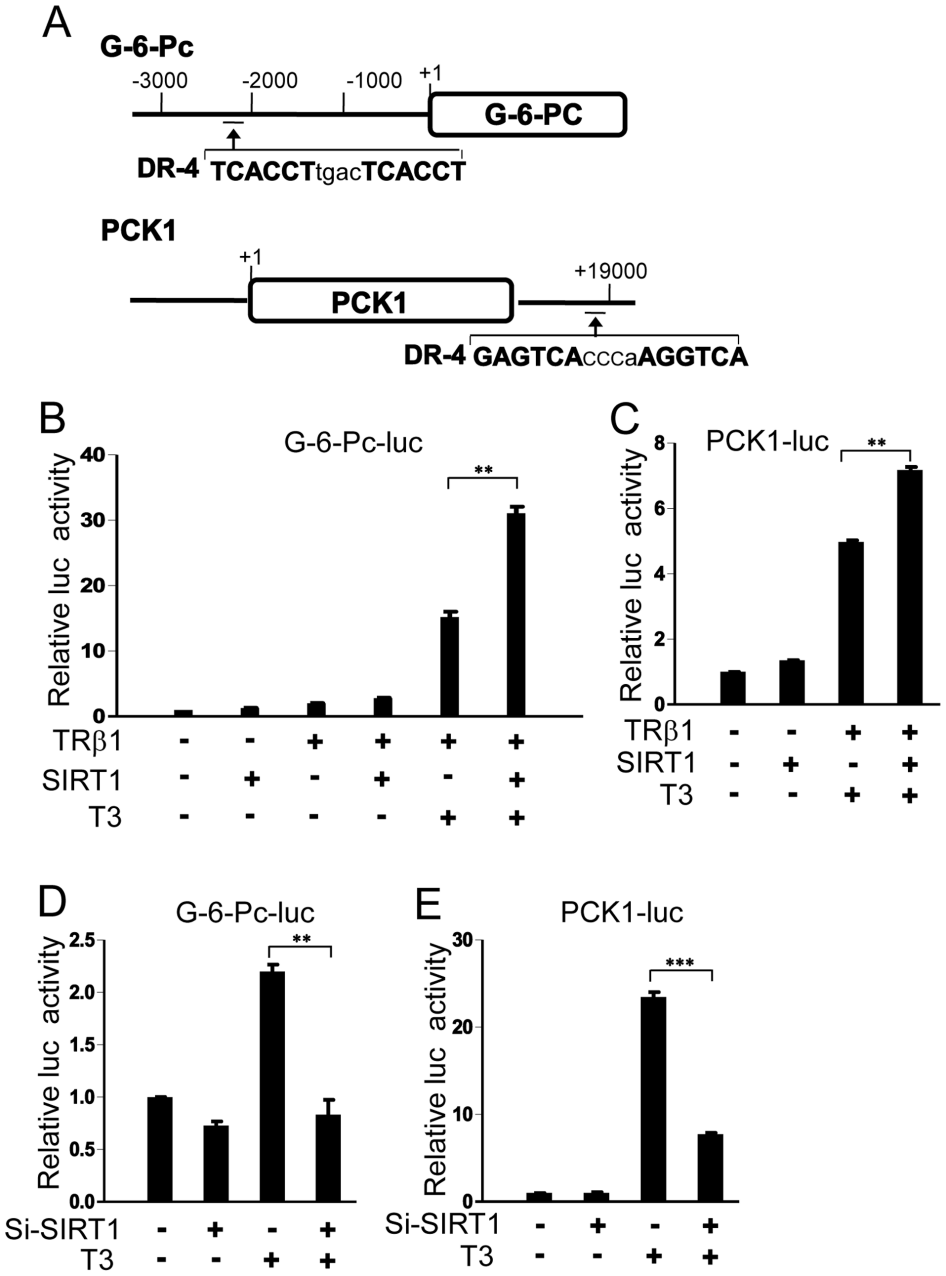
SIRT1 regulates TRβ1 target gene promoter activity. (**A**) Schematic representation of TRβ1 response regions (TREs) of TRβ1 target genes with sequences and positions of DR-4 sites for G-6-Pc gene and PCK1 gene. **(B–C)** Luciferase assays performed on extracts of 293T cells that were cotransfected with indicated reporters along with TRβ1 and SIRT1 expression vectors and treated +/− T_3_. **(D, E)** Luciferase assays performed on extracts of HepG2-TRβ1 cells transfected with indicated reporters and SIRT1 siRNA and treated +/− T_3_. The levels of luciferase activity were normalized to the lacZ expression. Data are representative of at least three independent experiments with similar results. All values represent mean ± SD of duplicate samples. **, *P* < 0.01; ***, *P* < 0.001.

To examine effects of SIRT1 on TRβ1 activity at native TREs in the G-6-Pc and PCK1 genes, we used computer aided analysis to localize potential regulatory elements in both loci. For the G-6-Pc gene, we detected a hitherto unknown DR-4 site approximately 2.3 kb upstream of the transcriptional initiation site (Fig. 8A). While previous studies suggested that the rodent PCK1 proximal promoter harbors a variant TRE [30]–[32], we were unable to locate functional TREs within the human PCK1 proximal promoter or demonstrate TRβ1 interaction with this region of DNA by transfection analysis or gel shift (not shown). However, we did localize a previously unknown non-canonical DR-4 site around 13KB downstream of the gene (Fig. 8A).

As expected, TRβ1 conferred T_3_ response upon reporters driven by the native G-6-Pc promoter and the PCK1 downstream TRE (Fig. 8B, C). This effect was enhanced by SIRT1 cotransfection (Fig. 8B, C) and, conversely, SIRT1 knockdown inhibited T_3_ response in both contexts (Fig. 8D, E). However, effects of SIRT1 overexpression and knockdown were more prominent at the G-6-Pc promoter versus the PCK1 downstream TRE (Fig. 8) and other native TREs (not shown), in parallel with strong SIRT1 requirements for T_3_ induction of the native G-6-Pc gene. T_3_ treatment and SIRT1 overexpression did not alter activity of the parental pGL4.23 reporter, indicating that effects were specific to G-6-Pc and PCK1 sequences (Fig. S4B).

To directly compare SIRT1 effects upon T_3_ response at TREs that localized to proximal promoters, analogous to G-6-Pc, and to rule out the possibility that the relative lack of a SIRT1 effect upon PCK1 was a consequence of the unusual source and composition of this element, we also assessed SIRT1 effects upon proximal promoters of other TRβ1 target genes (Fig. S4C, D). While SIRT1 potentiated T_3_ response at the SLC16A6 and MYH6 promoters, the extent of SIRT1 potentiation was promoter-specific with relatively modest effects at SLC16A6 and stronger effects upon MYH6 (Fig. S4C, D). Thus, SIRT1 enhances TRβ1 dependent T_3_ response in a promoter-specific action.

**Figure 9:**
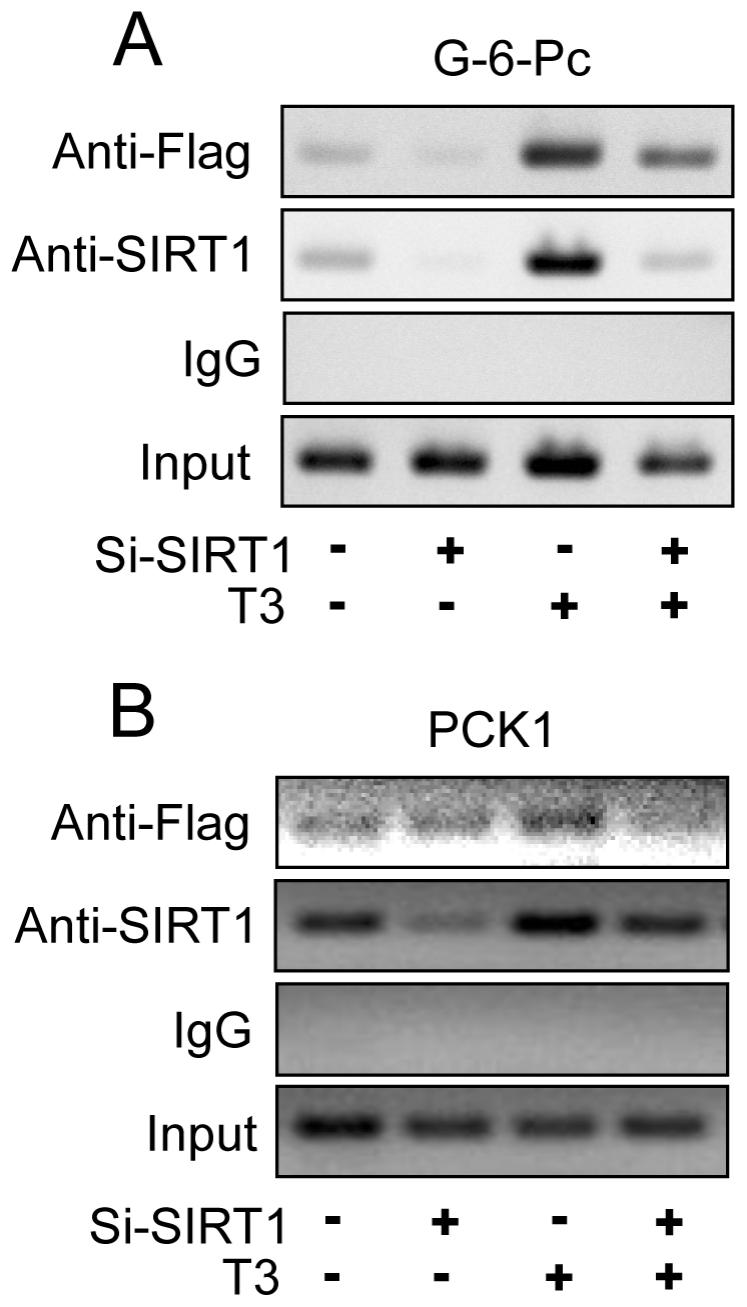
SIRT1 is recruited to TREs of TRβ1 target genes. ChIP assays performed in HepG2-TRβ cells treated +/− SIRT1 siRNA and T_3_. Antibodies used for immunoprecipitation were Flag, SIRT1 or IgG control. 10 % (v/v) of the supernatant was represented as ‘input’ chromatin prior to immunoprecipitation by antibodies.

To verify that TRβ1 and SIRT1 co-localized to the G-6-Pc and PCK1 TREs in cultured HepG2-TRβ1 cells, ChIP analysis was performed (Fig. 9A, B). We observed that TRβ1 (using an antibody to the Flag-tag at the N-terminus of TRβ1 expressed in these cells) and SIRT1 were present at both elements in the absence of hormone and SIRT1 siRNA knockdown reduced the amount of detectable SIRT1 protein at both TREs. Unlike many previously documented cases of hormone-independent interactions of TRs with TREs [1], T_3_ enhanced TRβ1 binding to the G-6-Pc promoter. SIRT1 recruitment to the G-6-Pc TRE also appeared hormone-dependent, even though TRβ1/SIRT1 interactions are unaffected by T_3_. More surprisingly, SIRT1 knockdown inhibited T_3_-dependent TRβ1 interactions with the G-6-Pc promoter. T_3_ weakly enhanced TRβ1 and SIRT1 binding to the PCK1 TRE and SIRT1 knockdown reversed the hormone-dependent component of this interaction. Thus, SIRT1 is recruited to TRE region of TRβ1 target genes and is required for T_3_-dependent association of TRβ1 with the G-6-Pc promoter and, to a lesser extent, the PCK1 downstream TRE.

### Drugs that Target SIRT1 Modulate TRβ1 Activity

**Figure 10:**
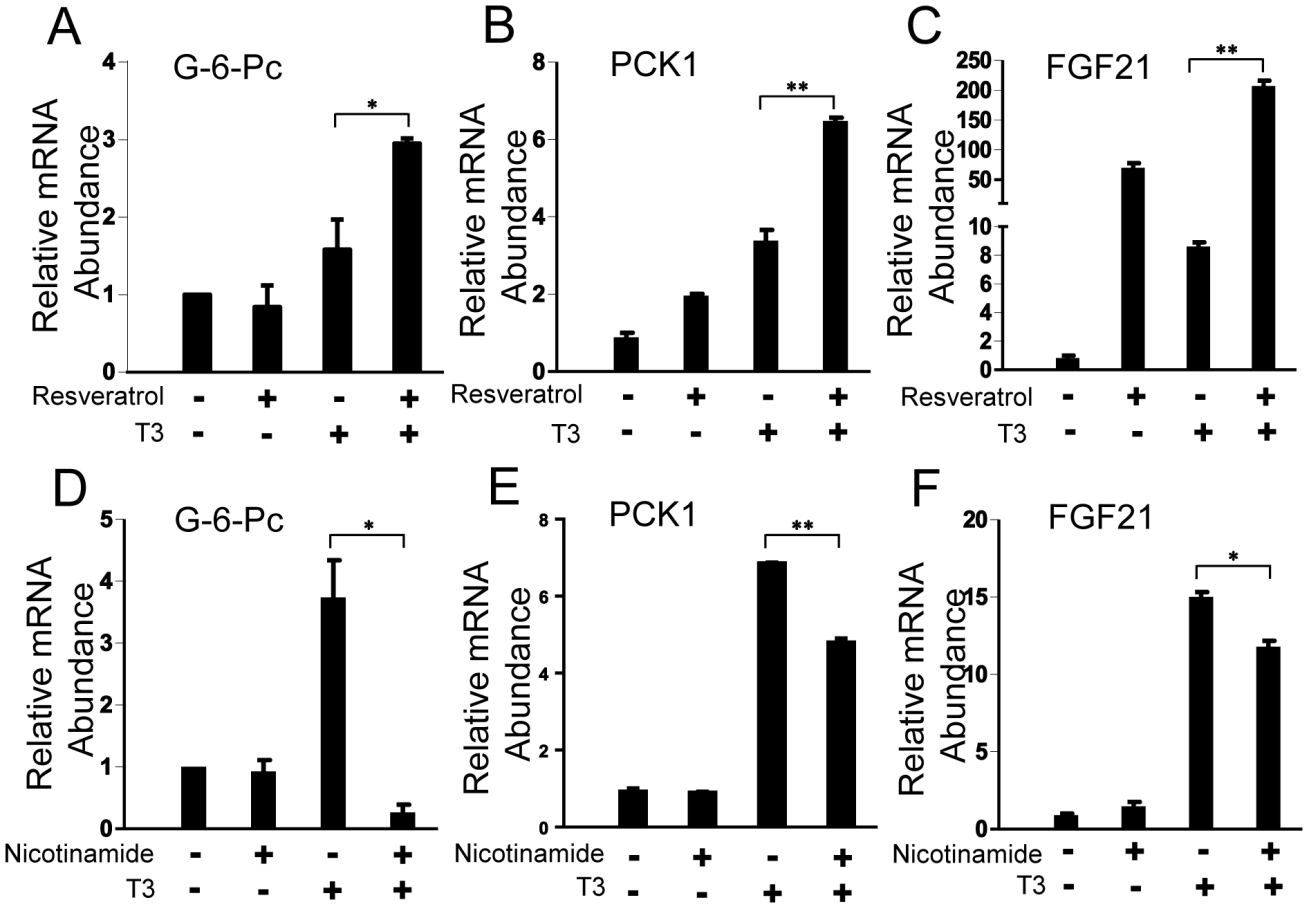
A Small Molecule SIRT1 activator and inhibitor regulate expression of TRβ1 target genes. **(A∼C)** qPCR analysis performed upon HepG2-TRβ cells treated with 100 µM resveratrol +/−T_3_. Genes are indicated at top. **(D∼F)** As for 10A–C, except that cells were treated with 10 mM nicotimamide instead of resveratrol. Data are representative of at least two independent experiments. All values represent the mean ± SD of duplicate samples. *, *P* < 0.05; **, *P* < 0.01.

Finally, we determined whether TRβ1 action at dual TRβ1/SIRT1 target genes was influenced by drugs that target the SIRT1 pathway. Treatment of HepG2-TRβ1 cells with resveratrol, an indirect activator of SIRT1, did not affect basal mRNA levels of G-6-Pc, but did enhance T_3_ response (Fig. 10A). In parallel, resveratrol enhanced basal PCK1 and FGF21 mRNA levels and also potentiated T_3_ response at both genes (Fig. 10B, C). Treatment of HepG2-TRβ1 cells with the SIRT1 inhibitor nicotinamide potently inhibited T_3_ induction of G-6-Pc (Fig. 10D), similar to effects of SIRT1 siRNA treatment. In parallel, nicotinamide modestly inhibited T_3_ response at PCK1 and FGF21 (Fig. 10E, F). Thus, chemical manipulation of SIRT1 influences T_3_ response and these SIRT1-dependent effects display similar gene context-specificity to that seen with SIRT1 siRNA.

## Discussion

In this study, we have investigated interactions of SIRT1 and TR signaling pathways. We hypothesized that PGC-1α and SIRT1 would cooperate to enhance TRβ1 activity in liver cells and, accordingly, we find that PGC-1α and SIRT1 synergize to potentiate T_3_ response at a TRE-dependent reporter, a very large enhancement of T_3_ activation in response to coregulator transfection compared with previous studies [9], [33]. Similar findings were recently reported by another investigator [24], who also demonstrated that SIRT1 promotes T_3_-dependent deacetylation of PGC-1α and that SIRT1 is required for optimal T_3_ response of endogenous TR-regulated genes in cultured liver cells, liver primary cultures and native rat liver. We have also obtained other evidence which suggests, however, that effects of SIRT1 are not completely dependent upon PGC-1α and that SIRT1 is also a direct TRβ1 coactivator. SIRT1 binds to TRβ1 in co-immunoprecipitation experiments and *in vitro* pulldowns and ChIP studies demonstrate colocalization of TRβ1 and SIRT1 at TREs located near target genes and similar results were also seen by Thakran and colleagues [24]. Furthermore, SIRT1 potentiates T_3_ response at a transfected reporter in the absence of exogenous PGC-1α and this effect is actually potentiated by knockdown of endogenous PGC-1α. Additionally, knockdown of endogenous PGC-1α does not diminish T_3_ response at two endogenous genes, opposite to effects of knockdown of SIRT1, implying that T_3_ response is PGC-1α independent in these conditions. Finally, SIRT1 overexpression triggers TRβ1 deacetylation and enhanced T_3_- and ubiquitin-dependent turnover of TRβ1. We acknowledge the possibility that SIRT1 could influence TRβ1 indirectly in the absence of PGC-1α, through actions upon another TR cofactor such as PGC-1β or other TR interacting proteins. However, correlation between SIRT1 enhancement of TRβ1 activity and SIRT1/TRβ1 interactions leads us to suggest that SIRT1 can modulate in two ways; indirectly, via potentiation of PGC-1α activity, and directly through TRβ1 contact.

While SIRT1 is a direct TR cofactor, requirements for SIRT1 in T_3_ response appear different from general TR coregulators such as the SRCs and TRAP220, which are needed for T_3_ activation of most TRβ1 target genes [3]. Instead, SIRT1 enhances TRβ1 activity in a strongly gene-specific manner. We found that SIRT1 knockdown abolished T_3_ induction of G-6-Pc, but only modestly inhibited T_3_ induction of the PCK1 and FGF21 genes, and left T_3_ response at HR and other genes completely unaffected. These results were internally consistent with experiments that utilized a small molecule activator (resveratrol) or an inhibitor (nicotinamide) of the SIRT1 pathway. Array based analysis of effects of SIRT1 knockdown in HepG2-TRβ cells confirmed that most T_3_ responses were independent of SIRT1, but also revealed that a small subset of TRβ1 target genes were strongly inhibited by SIRT1 knockdown. Studies of Thakran *et al.* are also indicative of gene specificity in TRβ1/SIRT1 cooperation [24]. Whereas T_3_ and resveratrol synergized to activate the pyruvate dehydrogenase kinase 4 (PDK4) gene in HepG2 cells, they only displayed modest additive effects at carnitine palmitoyl transferase 1a (CPT1a), and resveratrol did not enhance T_3_ response at other genes.

How does SIRT1 modulate TRβ1 activity? SIRT1 binds TRβ1 in a hormone-independent fashion. This is different from general coactivators such as the SRCs which interact with a ligand-dependent activation function (AF-2) in the receptor ligand binding domain [3], [33] and could imply that SIRT1 plays a distinct role from other TR coregulators. SIRT1 nevertheless largely influences T_3_ response, with only modest effects upon unliganded TRs in some conditions, implying that it must cooperate with factors that act upon T_3_-liganded TRs. There are also close parallels between effects of SIRT1 upon TRβ1 activity, acetylation state and turnover (this study) and previously reported effects of SIRT1 on LXRα [20]. In the latter study, the authors proposed that LXRs first induce the target gene in response to activating ligands and that SIRT1 subsequently deacetylates LXRα to trigger its ubiquitination and turnover, thereby allowing novel transcription complex formation. We have not investigated kinetics of changes in TRβ acetylation state through the transcription cycle, but it seems reasonable to suggest that SIRT1 could fulfill a similar function for TRs. TR acetylation is mediated by histone acetyl transferases (HATs) such as CBP/p300, among the first factors recruited to target genes after T_3_ binding [28]. We therefore suggest that SIRT1 must act at an essential step of TRβ1 activation that occurs after CBP/p300 dependent acetylation and that separate acetylation and deacetylation steps may be important components of the transcription cycle. To fully investigate this idea, it will be important to understand kinetics of recruitment of different cofactors that, respectively, acetylate and deacetylate TRs, the role of different TR acetylation sites in T_3_ response and the correlation of these events with TR acetylation status and transcriptional activity.

Data mentioned above describe general effects of SIRT1 upon TRβ activity, modification state and turnover, but it is hard to completely reconcile these effects with occasional strong gene-specific requirements for SIRT1 in T_3_ response. We instead propose that gene-specific effects of SIRT1 upon T_3_ response may be related to additional effects upon TRβ1 transcription complex formation. Three lines of evidence support this idea. T_3_ enhances SIRT1 recruitment to the G-6-Pc TRE. This is unexpected; TRβ1/SIRT1 interactions appear independent of hormone (Fig. 2). Further, T_3_ strongly enhances TRβ1 binding to the G-6-Pc promoter in cultured cells. This is also unusual; previous ChIP studies indicated that TRβ/TRE interactions are usually unaffected by T_3_. Finally, T_3_-dependent TRβ1 binding to the G-6-Pc promoter is potently inhibited by SIRT1 knockdown. We do not know of any other case in which TRβ1/TRE interactions in cultured cells are dependent upon a cofactor. Together, these observations suggest that T_3_ must trigger steps involved in TRβ/SIRT1 complex assembly on the G-6-Pc TRE. Interestingly, absolute requirements for SIRT1 in T_3_ response of G-6-Pc were recapitulated in transfections with a G-6-Pc promoter dependent reporter. Thus, information required for strong SIRT1 dependence of T_3_ response is located within this segment of DNA. It may be interesting to consider contributions of other TFs that bind the G-6-Pc promoter and cooperate with TRβ1 and/or SIRT1 in these effects and the possibility that TRβ1 acetylation inhibits TRβ binding in this context.

The possible physiologic significance of TRβ1/SIRT1 interactions is not completely clear. Given that PGC-1α is induced in fasting and that SIRT1 mediates beneficial effects of calorie restriction, it is reasonable to suggest that a TRβ1/PGC-1α/SIRT1 complex may be required for acute T_3_ response of genes involved in gluconeogenesis and fatty acid β-oxidation in liver [24]. Results of our experiments add another angle, strong SIRT1 requirements for responses of particular T_3_ regulated genes, including those seen at the G-6-Pc locus in this study and the PDK4 locus in another investigation [24] also raises the possibility that TRβ1/SIRT1 complex formation is an essential checkpoint for acute T_3_ regulation of particular subsets of genes *in vivo*. Another recent study reveals inverse correlation between thyroid hormone status and SIRT1 protein levels (but not mRNA levels) and SIRT1 activity in liver [41]. Given that physiological adaptation to fasting involves suppression of thyroid hormone actions, the authors suggest that increases in SIRT1 protein may be a specific response to reduced thyroid hormone status during caloric restriction. Our studies show that SIRT1 can exert PGC-1α independent effects on TRβ1, but we do not determine whether similar SIRT1 dependent effects on TRβ1 also occur in the presence of PGC-1α, whether these effects occur in mouse liver or precise physiologic conditions in which SIRT1 directly influences TRβ1. Thus, it is not obvious how molecular mechanisms described here may be involved in physiologic responses to fasting or hyperthyroidism. One interesting possibility, however, is that gene-specific SIRT1/TRβ1 interactions could preserve subsets of T_3_ regulated responses in conditions in which thyroid hormone levels and signaling pathways are broadly suppressed by starvation or fasting. We also note that thyroid hormone regulation of liver metabolic genes differs in different conditions; for example, thyroid hormone induction of gluconeogenic genes seen here in cell culture models can also be observed in mouse liver in some conditions but not others [42], [43]. It will be interesting to define the role of TRβ1/SIRT1 interactions in these phenomena. Given the general roles of SIRT1 in TRβ activity, acetylation and turnover that resemble those seen with LXRα and PPARγ [20], [44] and the additional gene-specific roles of TRβ/SIRT1 interactions, it will also be important to understand what types of effects are active in different types of physiological response to changes in TRβ1 or SIRT1 levels or actvity.

Regardless of the precise function of TRβ1/SIRT1 interactions, the fact that we can modulate T_3_ response at several endogenous genes with ligands that alter SIRT1 activity, resveratrol and nicotinamide, suggests that it may be possible to selectively manipulate subsets of key T_3_ responsive genes *in vivo* with combinations of thyromimetics and SIRT1 ligands. For example, SIRT1 activators could be used to enhance T_3_-dependent fatty acid oxidation in liver and a SIRT1 inhibitor could inhibit excessive gluconeogenesis associated with thyroid hormone excess states. These possibilities should be tested in animal models and could form the basis for novel therapeutic approaches to metabolic disease that employ SIRT1 modulators in combination with several thyromimetics that exhibit improved safety profiles relative to native thyroid hormones [34].

## Acknowledgments

We would like to thank Dr. Tadahiro Kitamura for expression vectors and adenoviruses.

## Supporting Information

Figure S1 **SIRT1 does not influence the acetylation of PGC-1α in transfected HepG2 cells.** Immunoprecipitation analysis of 293T cells transfected with expression vectors for PGC-1α, SIRT1 or both and treated +/− T_3_. PGC-1α was immunoprecipitated with anti- PGC-1α antibodies and precipitates were blotted with anti-acetyl-lysine, PGC-1α or SIRT1 antibodies. Acetylated PGC-1α levels relative to total TRβ1 were quantified by Phosphor Imager (right panel).
(TIF)

Figure S2 **Heatmap representation of TRβ1 target genes that are inhibited by SIRT1 knockdown.** T_3_-response was determined in the presence of Negative-control siRNA (NC-siRNA, column 1) and SIRT1 siRNA (Knock-down = KD, column 2) treatments through comparison against their respective vehicle control treatments. The specific effect of SIRT1 KD was determined through the comparison of effects of both SiRNA treatments in the absence of ligand (SIRT1-siRNA vs. NC-siRNA, lane 3), see Methods. Note that, in most instances, T_3_ responses are unaffected by SIRT1 ncokdown but that a subset of T_3_ responsive genes exhibit significant changes in response to SIRT1 knockdown. Further, while SIRT1 knockdown does influence target gene expression in the absence of T_3_, many effects of SIRT1 knockdown are specific to T_3_. The SIRT1/T_3_ dependent cluster shown in the main text is marked at right of the heatmap.
(TIF)

Figure S3 **The effect of PGC-1α knockdown upon expression of TRβ1 target genes.** qPCR analysis of HepG2-TRβ1 cells extracts treated +/− T_3_ and PGC-1α siRNA. G-6-Pc (A) and PCK1 (B). All values represent the mean ± SD of duplicate samples. **, *P* < 0.01; *, *P* < 0.05.
(TIF)

Figure S4 **SIRT1 differentially regulates the activity of alternate TRβ1 target gene promoters.** **(A)** Schematic representation of TREs of TRβ1 target genes with sequences and positions of DR-4 site (−309 ∼ −294) for SLC16A6 gene and DR-4 site (−148 ∼ −133) for MYH6 gene. **(B–D)** Luciferase assays performed on extracts of 293T cells that were cotransfected with indicated reporters along with TRβ1 and SIRT1 expression vectors and treated +/− T_3_. The levels of luciferase activity were normalized to the lacZ expression. All values represent mean ± SD of duplicate samples. **, P < 0.01; *, P < 0.05.
(TIF)
