# Supplementary figures and images for: SIRT1 is a Direct Coactivator of Thyroid Hormone Receptor β1 with Gene-Specific Actions
Source: PLoS One. 2013 Jul 26;8(7):e70097. doi: 10.1371/journal.pone.0070097 (PMC3724829; doi:10.1371/journal.pone.0070097)

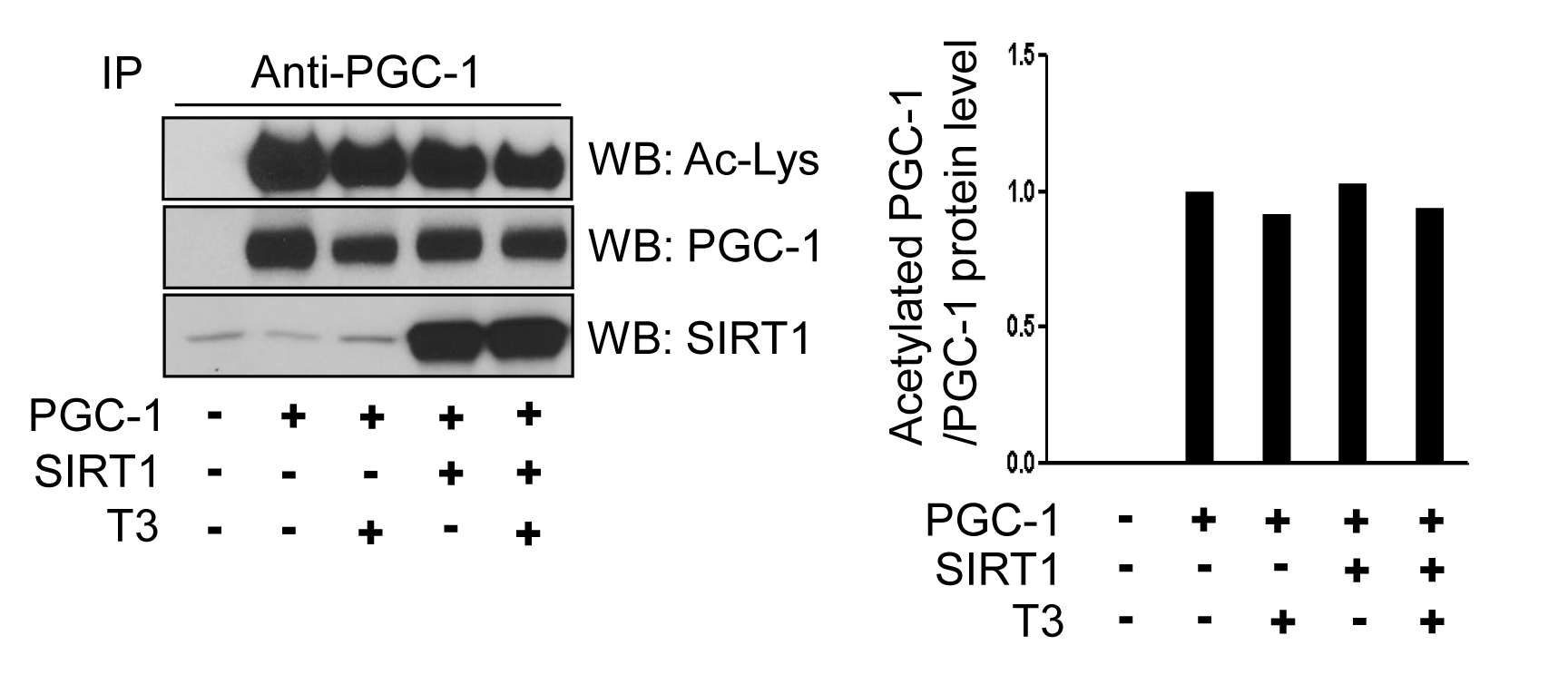

Supplement: Figure S1 — SIRT1 does not influence the acetylation of PGC-1α in transfected HepG2 cells. Immunoprecipitation analysis of 293T cells transfected with expression vectors for PGC-1α, SIRT1 or both and treated +/− T3. PGC-1α was immunoprecipitated with anti- PGC-1α antibodies and precipitates were blotted with anti-acetyl-lysine, PGC-1α or SIRT1 antibodies. Acetylated PGC-1α levels relative to total TRβ1 were quantified by Phosphor Imager (right panel). (TIF) [file pone.0070097.s001.tif]

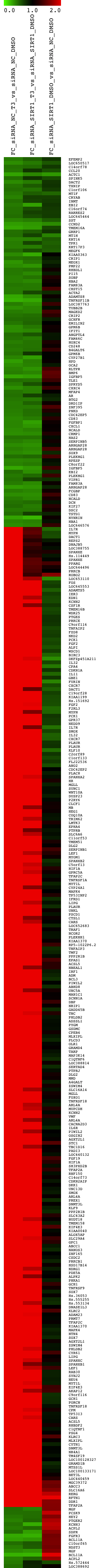

Supplement: Figure S2 — Heatmap representation of TRβ1 target genes that are inhibited by SIRT1 knockdown. T3-response was determined in the presence of Negative-control siRNA (NC-siRNA, column 1) and SIRT1 siRNA (Knock-down = KD, column 2) treatments through comparison against their respective vehicle control treatments. The specific effect of SIRT1 KD was determined through the comparison of effects of both SiRNA treatments in the absence of ligand (SIRT1-siRNA vs. NC-siRNA, lane 3), see Methods. Note that, in most instances, T3 responses are unaffected by SIRT1 ncokdown but that a subset of T3 responsive genes exhibit significant changes in response to SIRT1 knockdown. Further, while SIRT1 knockdown does influence target gene expression in the absence of T3, many effects of SIRT1 knockdown are specific to T3. The SIRT1/T3 dependent cluster shown in the main text is marked at right of the heatmap. (TIF) [file pone.0070097.s002.tif]

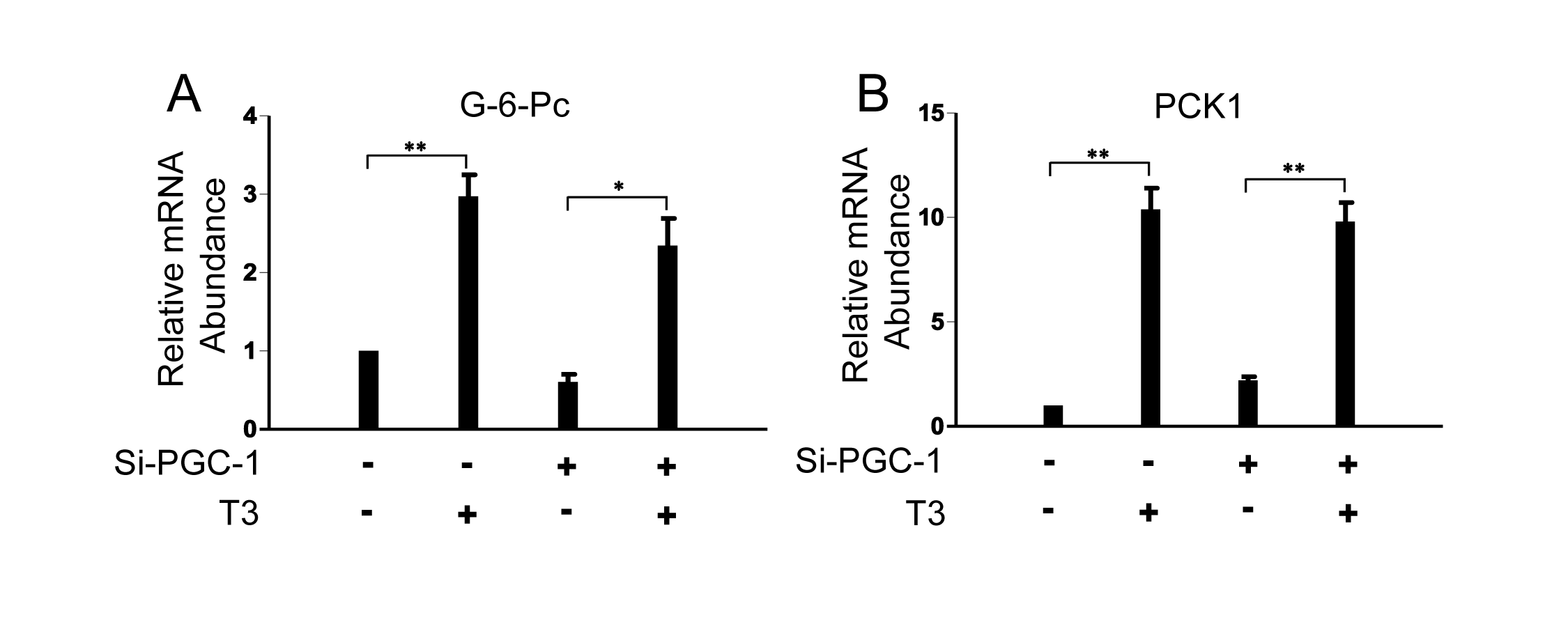

Supplement: Figure S3 — The effect of PGC-1α knockdown upon expression of TRβ1 target genes. qPCR analysis of HepG2-TRβ1 cells extracts treated +/− T3 and PGC-1α siRNA. G-6-Pc (A) and PCK1 (B). All values represent the mean ± SD of duplicate samples. **, P < 0.01; *, P < 0.05. (TIF) [file pone.0070097.s003.tif]

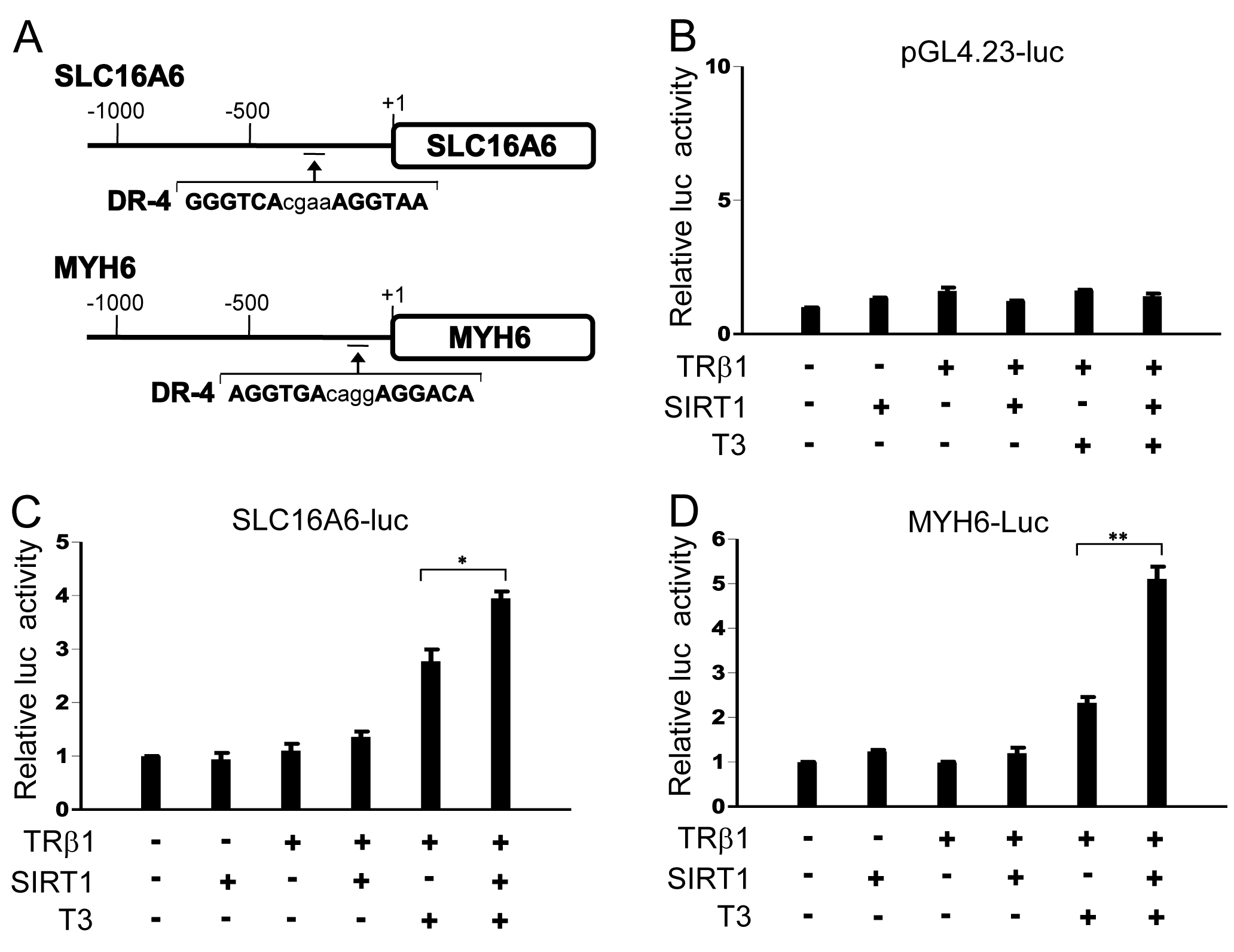

Supplement: Figure S4 — SIRT1 differentially regulates the activity of alternate TRβ1 target gene promoters. (A) Schematic representation of TREs of TRβ1 target genes with sequences and positions of DR-4 site (−309 ∼ −294) for SLC16A6 gene and DR-4 site (−148 ∼ −133) for MYH6 gene. (B–D) Luciferase assays performed on extracts of 293T cells that were cotransfected with indicated reporters along with TRβ1 and SIRT1 expression vectors and treated +/− T3. The levels of luciferase activity were normalized to the lacZ expression. All values represent mean ± SD of duplicate samples. **, P < 0.01; *, P < 0.05. (TIF) [file pone.0070097.s004.tif]
